# Combined α_2_- and D_2_-receptor blockade activates noradrenergic and dopaminergic neurons, but extracellular dopamine in the prefrontal cortex is determined by uptake and release from noradrenergic terminals

**DOI:** 10.3389/fphar.2023.1238115

**Published:** 2023-08-23

**Authors:** Claudia Sagheddu, Paola Devoto, Sonia Aroni, Pierluigi Saba, Marco Pistis, Gian Luigi Gessa

**Affiliations:** ^1^ Department of Biomedical Sciences, University of Cagliari, Cagliari, Italy; ^2^ The Guy Everett Laboratory, University of Cagliari, Cagliari, Italy; ^3^ Neuroscience Institute of CNR, Cagliari, Italy; ^4^ Unit of Clinical Pharmacology, University Hospital of Cagliari, Cagliari, Italy

**Keywords:** antipsychotics, locus coeruleus, ventral tegmental area, electrophysiology, microdialysis, rat

## Abstract

Experimental and clinical evidence indicates a deficit of release and function of dopamine in schizophrenia and suggests that α_2_-adrenoceptor antagonists rescue dopamine deficit and improve the antipsychotic efficacy of D_2_-receptor antagonists. In anesthetized male rats, we investigated how the blockade of α_2_- and D_2_-receptors by atipamezole and raclopride, respectively, modified the firing of noradrenergic neurons in the locus coeruleus (LC) and dopaminergic neurons in the ventral tegmental area (VTA). In freely moving rats, we studied how atipamezole and raclopride modified extracellular noradrenaline, dopamine, and DOPAC levels in the medial prefrontal cortex (mPFC) through microdialysis. When administered alone, atipamezole activated LC noradrenaline but not VTA dopamine cell firing. Combined with raclopride, atipamezole activated dopamine cell firing above the level produced by raclopride. Atipamezole increased extracellular dopamine to the same level, whether administered alone or combined with raclopride. In the presence of the noradrenaline transporter (NET) inhibitor, atipamezole combined with raclopride increased extracellular dopamine beyond the level produced by either compound administered alone. The results suggest that a) the D_2_-autoreceptor blockade is required for LC noradrenaline to activate VTA cell firing; b) the level of dopamine released from dopaminergic terminals is determined by NET; c) the elevation of extracellular dopamine levels in the mPFC is the resultant of dopamine uptake and release from noradrenergic terminals, independent of dopaminergic cell firing and release; and d) LC noradrenergic neurons are an important target for treatments to improve the prefrontal deficit of dopamine in neuropsychiatric pathologies.

## Introduction

During the last two decades, α_2_-adrenoceptor antagonists have been proposed in adjunctive therapy to improve the efficacy of antipsychotics in schizophrenia (Hertel et al., 1999a; Marcus et al., 2010; Brosda et al., 2014), to potentiate the antidepressant effect of noradrenaline and serotonin uptake inhibitors (Pozzi et al., 1994; Invernizzi and Garattini, 2004; Ortega et al., 2010), to alleviate the extrapyramidal effects of neuroleptics (Henry et al., 1999; Invernizzi et al., 2003; Imaki et al., 2009), to potentiate the anti-parkinsonian effect of dopamine agonists (Henry et al., 1999; Haapalinna et al., 2003; Savola et al., 2003), and to alleviate L-DOPA-induced dyskinesia (Rascol et al., 2001; Ostock et al., 2015). The add-on strategy of α_2_-adrenoceptor antagonists in the treatment of schizophrenia has been motivated by the awareness that the prototype atypical antipsychotic, clozapine, is a potent α_2_-adrenoceptor antagonist and that its ability to block both α_2_-adrenoceptors and dopaminergic D_2_-receptors plays an important role in its atypical actions, including its efficacy against negative symptoms in schizophrenia, antidepressant activity, and low propensity to induce extrapyramidal effects (Gerlach, 1991; Breier, 1994; Litman et al., 1996; Kapur and Remington, 2001; Kalkman and Loetscher, 2003; Elsworth et al., 2008; Khokhar et al., 2018; Gammon et al., 2021). The ability of adjunctive treatment with α_2_-adrenoceptor antagonists to improve the therapeutic efficacy of antipsychotic drugs has been attributed to the facilitation of dopamine transmission in the prefrontal cortex (PFC) and the striatum (Svensson, 2003; Masana et al., 2011).

To explain how α_2_-adrenoceptor antagonists facilitate dopamine transmission in PFC, it has been suggested that they increase dopamine output by activating dopaminergic cells in the ventral tegmental area (VTA) via noradrenaline release from locus coeruleus (LC) neurons (Park et al., 2017). Alternatively, α_2_-adrenoceptor antagonists would increase dopamine output independently via cell firing, i.e., at the nerve terminal level, either by removing a tonic inhibitory α_2_-mediated control by noradrenaline on dopamine release (Hertel et al., 1999b) or by increasing extracellular noradrenaline, which would reduce dopamine clearance from the extracellular space by competing for the same transporter (Carboni et al., 1990; Pozzi et al., 1994; Gresch et al., 1995; Hertel et al., 1999b). At variance with these interpretations, previous results from our laboratory suggest that noradrenergic terminals are the primary source of dopamine measured by microdialysis in the medial PFC (mPFC) (Devoto and Flore, 2006). Supporting our hypothesis, the extracellular dopamine concentration in the occipital and cerebellar cortexes, where dopamine innervation is scarce or absent, was found to be of the same magnitude as in the mPFC, which is densely innervated by dopaminergic terminals, consistent with a comparable noradrenergic innervation in the three regions (Devoto et al., 2001; Devoto et al., 2003). Consistently, clozapine was found to produce a comparable elevation of extracellular dopamine in mPFC as in the occipital cortex (Devoto et al., 2001; Devoto et al., 2003; Devoto et al., 2019).

Moreover, noradrenergic, but not dopaminergic, agonists and antagonists were found to modify the extracellular dopamine level in mPFC (Devoto and Flore, 2006), while noradrenergic denervation suppressed α_2_-receptor-mediated dopamine release (Devoto et al., 2019).

While an increasing number of clinical studies support the validity of the adjunctive α_2_-adrenergic blockade to enhance the antipsychotic effect of typical and atypical antipsychotics (Wadenberg et al., 2007; Brosda et al., 2014; Langer, 2015), how exactly this strategy works remains to be elucidated.

Because of these differing interpretations, the present study was designed to clarify how the separate or combined α_2_- and D_2_-receptor blockade by atipamezole and raclopride, respectively, modifies the electrical activity of noradrenergic neurons in LC and dopaminergic neurons in VTA. Moreover, we analyzed how atipamezole, raclopride, and their combination modify the release and the extracellular levels of noradrenaline, dopamine, and DOPAC in mPFC by microdialysis.

## Methods and materials

### Subjects

Male Sprague–Dawley rats (Charles River, Italy), weighing 250–400 g, were group-housed and maintained under a regular 12:12 h light/dark cycle in temperature- and humidity-controlled facilities with food and water available *ad libitum*. The experimental protocols were conducted to minimize pain and suffering and to reduce the number of animals used. The study involving the animals were reviewed and approved by Dr. V. U. Santucci, “Direzione generale della sanità animale e dei farmaci veterinari, ufficio 6,” at the Italian Ministry of Health (Aut. No. 611/2017-PR) and was carried out in accordance with the European Directive on the protection of animals used for scientific purposes (2010/63/EU).

### Drugs

Drugs were dissolved in sterile distilled water or saline and administered in a volume of 1 mL/kg. Atipamezole hydrochloride [5-(2-ethyl-2,3-dihydro-1H-inden-2-yl)-1H-imidazole hydrochloride, CAS No. 104075-48-1; Orion Pharma] was administered at 3 mg/kg i.p. or up to 0.5 mg/kg i.v. for microdialysis or electrophysiological experiments, respectively.

S(−)-raclopride (+)-tartrate salt [3,5-dichloro-N-(1-ethylpyrrolidin-2-ylmethyl)-2-hydroxy-6- methoxybenzamide (+)- tartrate salt, CAS No. 98185-20-7; Sigma-Aldrich] was administered at 0.5 mg/kg i.p. or up to 0.025 mg/kg i.v. for microdialysis or electrophysiological experiments, respectively. Prazosin [1-(4-amino-6,7-dimethoxy-2-quinazolinyl)-4-(2-furanylcarbonyl)piperazine hydrochloride, CAS No. 19237-84-4; Tocris] was administered at 1 mg/kg i.v. for electrophysiological experiments. Nisoxetine hydrochloride [(±)-γ-(2-methoxyphenoxy)-N-methyl benzene propanamine hydrochloride, CAS No. 57754-86-6; Tocris] was administered at 3 mg/kg i.p. for microdialysis experiments.

### *In vivo* single-unit extracellular recordings

The rats were anesthetized with chloral hydrate (400 mg/kg, i.p.), and their femoral vein was cannulated for the i.v. administration of pharmacological agents. The rats were placed in a stereotaxic frame (Kopf, Tujunga, CA, United States) with their body temperature maintained at 37°C ± 1°C using a heating pad. According to the stereotaxic rat brain atlas by Paxinos and Watson (2007) (The Rat Brain, 2022), the recording electrode was placed above the LC (9.5–10.0 mm posterior to bregma, 1.0–1.2 mm lateral, and 5.5–6.5 mm from the cerebellar cortex) or the VTA (5.6–6.0 mm posterior to bregma, 0.4–0.6 mm lateral, and 7.0–8.0 mm from the cortical surface). The single-unit activity of neurons was recorded extracellularly (bandpass filter: 0.1–10,000 Hz) using glass micropipettes filled with 2% pontamine sky blue dissolved in 0.5 M sodium acetate. Individual action potentials were isolated and amplified using a CP511 AC Amplifier (Grass Instruments Co., US) and displayed on a digital storage oscilloscope (TDS 3012, Tektronix, Marlow, United Kingdom). Experiments were sampled using Spike2 software in a computer connected to the CED 1401 interface (Cambridge Electronic Design, Cambridge, United Kingdom). Spontaneously active noradrenergic neurons were identified using the following criteria: regular firing rate (0.5–5.0 Hz) and positive action potential of long duration (3–4 ms), exhibiting a brisk excitatory response to a nociceptive pinch of the contralateral hind paw (Cedarbaum and Aghajanian, 1977; Muntoni et al., 2006; Guiard et al., 2008). Spontaneously active dopamine neurons were identified using the following criteria: ≤10 Hz firing rate and positive triphasic action potentials (duration ≥2.5 ms). Bursts occurred as groups of two or more action potentials at an interspike interval of <80 ms and terminated at >160 ms intervals (Grace and Bunney, 1984).

The baseline spontaneous firing rate was recorded for 3–5 min; the drugs were administered at 120 s intervals.

### Microdialysis

The rats were deeply anaesthetized with Equithesin (0.97 g pentobarbital, 2.1 g MgSO_4_, 4.25 g chloral hydrate, 42.8 mL propylene glycol, and 11.5 mL 90% ethanol in 100 mL; 5 mL/kg, i.p.) and stereotaxically implanted with vertical microdialysis probes (membrane AN69-HF, Hospal-Dasco, Bologna, Italy; cutoff 40,000 Da) in the mPFC (3 mm active membrane length; AP +3.0, L ± 0.6, and V −6.5 mm from the bregma), according to Paxinos and Watson (The Rat Brain, 2022). The day after probe implantation, artificial cerebrospinal fluid (147 mM NaCl, 4 mM KCl, 1.5 mM CaCl_2_, and 1 mM MgCl_2_; pH 6–6.5) was pumped using a CMA/100 microinjection pump (Carnegie Medicine, Stockholm, Sweden) through the dialysis probes at a constant rate of 1.1 μL/min in freely moving animals. Dialysate samples were collected every 20 min and immediately injected using HPLC. Drugs were administered after stable extracellular levels were obtained, i.e., three consecutive samples with a variance not exceeding 15%. Noradrenaline, dopamine, and DOPAC were simultaneously analyzed using HPLC with electrochemical detection using HPLC systems equipped with 3.0 × 150 mm C18 (3.5 µ) Symmetry columns (Waters, Milan, Italy), maintained at 40°C using Series 1100 thermostats (Agilent Technologies, Waldbronn, Germany) and ESA Coulochem II detectors (Chelmsford, MA, United States). The mobile phase was 80 mM Na_2_HPO_4_, 0.27 mM EDTA, 0.6 mM sodium octyl sulfate, 7% methanol, and 4% acetonitrile, adjusted to pH 2.4 with H_3_PO_4_, delivered at 0.3 mL/min; the Coulochem analytical cell first electrode was set at +200 mV and the second was set at −200 mV. Quantification was performed by recording the second electrode signal. Under these conditions, the noradrenaline and dopamine detection limit (signal-to-noise ratio: 3:1) was 0.3 pg per injection on the column. On the completion of testing, the rats were euthanized by an Equithesin overdose; their brains were removed and sectioned using a cryostat (Leica CM3050 S) into 40-µm-thick coronal slices to verify the locations of dialysis probes.

### Data analysis and statistics

In microdialysis experiments, the average of three basal samples was considered 100% for the calculation of drug-induced variations. For electrophysiology, only one cell per rat was recorded. Changes in the firing rate were calculated by averaging the effects of the drugs for the 2-min period following drug administration and comparing them with the mean of the pre-drug baseline. All statistical analyses were performed using GraphPad Prism Software (La Jolla, California, United States). Data were analyzed by one-way or two-way repeated measures (RM) ANOVA. The *post hoc* multiple comparison test was carried out using Dunnett’s or Tukey’s test, as appropriate. *p* <0.05 was considered significant.

## Results

### Electrophysiology

**FIGURE 1:**
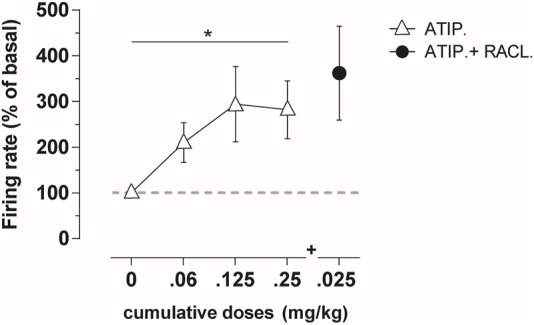
Average percentage of the firing frequency in LC noradrenergic neurons (*n* = 7) following cumulative atipamezole (0.06–0.25 mg/kg; white triangles) and adjunctive raclopride (0.025 mg/kg; black dot) i.v. administration in anesthetized rats. Atipamezole produced a significant increase in the firing rate of noradrenergic neurons, with no additional effect by the subsequent raclopride administration. Data are shown as % mean ± SEM of the basal value. *p <0.05 RM one-way ANOVA of the atipamezole dose curve.

The intravenous administration of atipamezole (at cumulative doses of 0.06–0.25 mg/kg i.v.; *n* = 7) produced a dose-dependent increase in the firing rate of LC noradrenergic neurons (Figure 1; RM one-way ANOVA: F(1,712, 10,27) = 4.931; *p* = 0.035).

**FIGURE 2:**
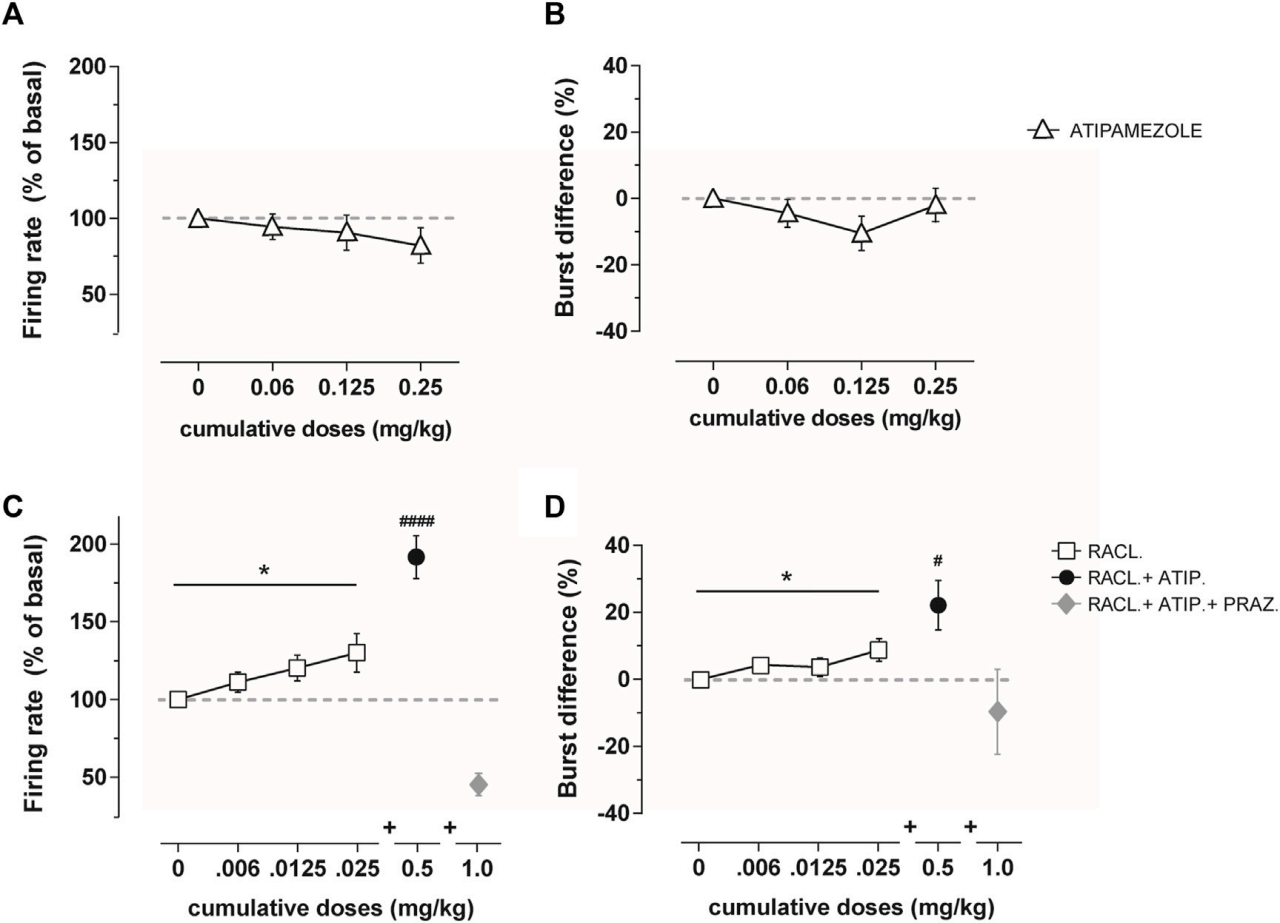
Average percentage of the firing frequency **(A)** and burst difference **(B)** of VTA dopamine neurons (*n* = 6–7) following cumulative atipamezole i.v. administration (0.06–0.25 mg/kg; white triangles) in anesthetized rats. Average percentage of the firing frequency **(C)** and burst difference **(D)** of VTA dopamine neurons following cumulative raclopride (*n* = 10, 0.006–0.025 mg/kg; white squares), adjunctive atipamezole (*n* = 10, 0.5 mg/kg; black dot), and adjunctive prazosin (*n* = 4, 1.0 mg/kg; gray diamond) administration in anesthetized rats. Atipamezole alone did not change the firing activity of dopamine neurons, whereas it increased the effect when administered after raclopride. Data are shown as % mean ± SEM of the basal value. *p <0.05 RM one-way ANOVA of the raclopride dose curve. #*p* <0.05 and #*p* <0.0001 RM one-way ANOVA of atipamezole + raclopride.

On the other hand, atipamezole at the same cumulative dose modified neither the firing rate (RM one-way ANOVA: F(2,162, 12,97) = 1.242; *p* = 0.32; fig. 2A) nor the bursting activity (RM one-way ANOVA: F(1,854, 11,12) = 1.43; *p* = 0.27; fig. 2B) of dopamine neurons in VTA (*n* = 6–7).

Adjunct to raclopride, atipamezole at the dose of 0.5 mg/kg (*n* = 10) produced an additional increase in the firing rate (RM one-way ANOVA F(2,075, 18,67) = 23.64; *p* <0.0001; Figure 2C) and bursting activity (RM one-way ANOVA: F(2,259, 11,33) = 4.82; *p* = 0.043; Figure 2D) above the maximal effect produced by raclopride (RM one-way ANOVA, firing rate: F(1,451, 13,06) = 4.473; *p* = 0.042; bursting activity: F(1,968, 17,71) = 4.09; *p* = 0.035; Figures 2C,D). The subsequent administration of the α1-adrenoceptor antagonist prazosin (1 mg/kg i.v.; *n* = 4) reversed the activation produced by the combined administration of atipamezole and raclopride (Figures 2C,D).

Raclopride (0.025 mg/kg i.v.) failed to modify the atipamezole-induced activity of LC noradrenergic cell firing (Figure 1).

### Microdialysis

**FIGURE 3:**
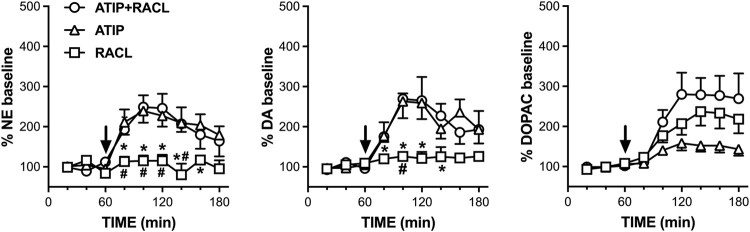
Effect of raclopride (0.5 mg/kg i.p., *n* = 4), atipamezole (3 mg/kg i.p., *n* = 7), and their combination (*n* = 4) on extracellular noradrenaline, dopamine, and DOPAC levels in mPFC of the rats, respectively. Data are expressed as % mean ± SEM of the basal value. Drugs were administered at T = 60 min, as indicated by the arrows. *p <0.05 vs*.* atipamezole; #*p* <0.05 vs*.* atipamezole + raclopride.

The effect of the intraperitoneal administration of atipamezole (3 mg/kg; *n* = 7), raclopride (0.5 mg/kg; *n* = 4), and their combination (*n* = 4) was analyzed by microdialysis in mPFC in freely moving rats (Figure 3). The baseline dialysate levels of noradrenaline, dopamine, and DOPAC were 3.0 ± 0.4 pg, 1.7 ± 0.2 pg, and 216 ± 25.4 pg, respectively. Values are expressed as mean ± SEM in pg/20 µL dialysate samples.

Atipamezole increased the extracellular noradrenaline levels to a maximum of 240% of the basal values (RM one-way ANOVA: F(1.906, 11.43) = 10.99; *p* = 0.002), while raclopride failed to modify the extracellular dopamine level (F(1.816, 5.448) = 1.533; *p* = 0.293). The combined administration of atipamezole and raclopride increased extracellular noradrenaline to the same level (250% of the baseline; RM one-way ANOVA: F(1.663, 4.989) = 8.277; *p* = 0.028) as that elicited by atipamezole alone. Two-way RM ANOVA evidenced a significant treatment effect (F(2, 12) = 6.93; *p* = 0.010). The effect of raclopride was significantly different from that of atipamezole at time points from T80 to T160 min and from that of atipamezole plus raclopride at time points from T80 to T140 min; no difference was found between the effects of atipamezole alone and atipamezole combined with raclopride (Šídák’s multiple comparison *post hoc* test).

The effect of atipamezole, raclopride, and their combination on extracellular dopamine reproduced those on extracellular noradrenaline. Thus, atipamezole increased extracellular dopamine to the same level (250% of the baseline) when administered alone (RM one-way ANOVA: F(2.184, 13.10) = 10.60; *p* = 0.001) as well as combined with raclopride (RM one-way ANOVA: F(1.762, 5.286) = 12.49; *p* = 0.011), while raclopride alone was ineffective (RM one-way ANOVA: F(1.366, 4.097) = 0.9247; *p* = 0.425).

Two-way repeated measures ANOVA evidenced a significant treatment effect (F(2, 12) = 4.95; *p* = 0.027). The effect of raclopride was significantly different from that of atipamezole at time points T80, T100, T120, and T160 min and from that of atipamezole plus raclopride at time point T100 min; no difference was found between the effects of atipamezole alone or in association with raclopride (Šídák’s multiple comparison *post hoc* test).

Extracellular DOPAC levels were increased to 160, 240, and 280% of the basal values by atipamezole, raclopride, and their association, respectively (RM one-way ANOVA, atipamezole: F(1.542, 9.251) = 8.723, *p* = 0.010; raclopride: F(1.223, 3.668) = 12.54, *p* = 0.026; and atipamezole + raclopride: F(1.295, 3.886) = 10.62 , *p* = 0.030). Two-way repeated measures ANOVA indicated a significant effect of the treatment (F(2, 12) = 4.37; *p* = 0.037), but no difference was found by Šídák’s *post hoc* test.

To verify whether the uptake into the noradrenergic terminal limited the elevation of extracellular dopamine elicited by the combination of atipamezole and raclopride, the effect of atipamezole alone and combined with raclopride was analyzed after the blockade of the noradrenergic transporter (NET) with nisoxetine (3 mg/kg, i.p.).

**FIGURE 4:**
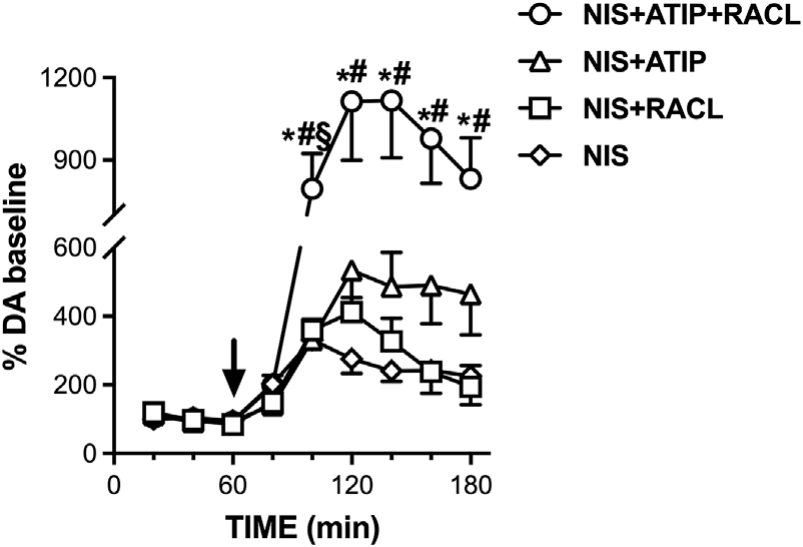
Effect of nisoxetine (3 mg/kg i.p., *n* = 6) alone and combined with atipamezole (3 mg/kg i.p., *n* = 5), raclopride (0.5 mg/kg i.p., *n* = 6), and their combination (*n* = 11) on extracellular dopamine levels in mPFC of the rats. Data are expressed as % mean ± SEM of the basal value. Drugs were administered at T = 60 min, as indicated by the arrow. *p <0.05 vs*.* nisoxetine; #*p* <0.05 vs*.* nisoxetine + raclopride. §*p* <0.05 vs*.* nisoxetine + atipamezole.

Figure 4 shows that nisoxetine administered alone (*n* = 6) increased the extracellular dopamine level to 330% of the baseline (RM one-way ANOVA: F(1.701, 8.507) = 14.99; *p* = 0.002). Raclopride coadministered with nisoxetine (*n* = 6) increased the extracellular dopamine level to 410% (RM one-way ANOVA: F(2.443, 12.21) = 17.04; *p* = 0.0002) and with atipamezole (*n* = 5) to 530% of the baseline (RM one-way ANOVA: F(1.136, 5.681) = 11.61; *p* = 0.014).

Crucially, in the presence of NET blockade, the combination of atipamezole and raclopride (*n* = 11) increased the extracellular dopamine level to more than ten times the baseline. Two-way repeated measures ANOVA evidenced a significant effect of the treatment (F(3, 24) = 6.50; *p* = 0.002), due to the difference between nisoxetine + atipamezole + raclopride co-administration with respect to the other treatments, as no difference was found between nisoxetine alone and nisoxetine–raclopride or nisoxetine–atipamezole combination (Šídák’s multiple comparison *post hoc* test).

## Discussion

Consistent with its ability to inhibit α_2_-adrenoceptors, systemic atipamezole activated the firing of noradrenergic neurons in LC and increased extracellular noradrenaline levels in mPFC. Moreover, atipamezole also increased the extracellular dopamine level in mPFC, an effect previously observed with other α_2_-adrenoceptor antagonists and attributed to the stimulation of dopamine neurons in VTA by noradrenaline released by noradrenergic terminals (Gresch et al., 1995; Gobert et al., 1997; Ihalainen and Tanila, 2002).

Against this hypothesis, atipamezole, administered alone, did not stimulate dopaminergic neurons in VTA, yet it increased the extracellular dopamine level in mPFC. These considerations support the hypothesis that noradrenergic terminals are the primary source of α_2_-adrenoceptor-mediated dopamine release in mPFC. A previous observation that the α_2_-adrenoceptor-mediated elevation of extracellular dopamine in mPFC was prevented by noradrenergic denervation (Mejias-Aponte, 2016; Devoto et al., 2019; Devoto et al., 2020) is consistent with this interpretation.

The discrepant results observed with other α_2_-adrenoceptor antagonists, such as yohimbine, compound RS 79948, and idazoxan, should be analyzed further on the fact that idazoxan also blocks imidazoline receptors (MacKinnon et al., 1989) and acts as an agonist at 5-HTA receptors (Newman-Tancredi et al., 1998), while yohimbine and RS 79948 also block D_2_-receptors (Scatton et al., 1980; Millan et al., 2000; Frau et al., 2022).

Notably, while atipamezole was ineffective when administered alone, adjunct to raclopride, activated dopaminergic cell firing in VTA further above the level was produced by raclopride alone.

The results revealed that the D_2_- and α_2_-receptor blockade is required for atipamezole to activate dopamine cell firing and suggested that the noradrenergic-induced activation of VTA dopamine neurons is contrasted by dopamine released from dopamine cell dendrites or co-released with noradrenaline from noradrenergic terminals, acting on D_2_-autoreceptors.

A major outcome of this study was that the activation of dopamine cell firing by the combined α_2_- and D_2_-receptor blockade increased extracellular DOPAC but, surprisingly, failed to increase extracellular dopamine above the level produced by atipamezole administered alone, while the rise of DOPAC was correlated with dopamine cell firing and reflected the activity of the dopamine transporter (DAT). Changes in extracellular DOPAC were correlated with the electrical activity of dopamine neurons, which is in line with the notion that DOPAC represents the intracellular oxidation of dopamine presynaptically recaptured by DAT other than the oxidation of newly synthetized dopamine (Wallace and Traeger, 2012).

On the other hand, in contrast to what could be expected from the electrical activity of dopamine neurons, atipamezole produced the same increase in extracellular dopamine levels, whether administered alone or in combination with raclopride. Burst activation of dopamine neurons by electrical stimulation of VTA has been shown to produce a long lasting availability of released dopamine in cortical (Au-Young et al., 1999) and subcortical areas (Lohani et al., 2018), an effect attributed to DAT internalization (Lohani et al., 2018). Electrical stimulation of VTA might include concurrent activation of noradrenergic fibers projecting in the mPFC, such as the medial forebrain bundle. Raclopride, like other typical antipsychotics, selectively activates dopamine cell firing in VTA but does not increase dopamine levels in cortical areas. To explain this dissociation between neuron firing and extracellular dopamine elevation, we postulate that dopamine released from dopaminergic terminals in mPFC is partly recaptured by DAT to be oxidized to DOPAC, while the majority escapes the synapse to be taken up by NET into noradrenergic terminals. Direct evidence for this hypothesis was the rapid, massive elevation of extracellular dopamine produced by the combination of atipamezole and raclopride in the presence of the inhibition of NET with nisoxetine. Remarkably, the magnitude of dopamine rises after the combination of atipamezole and raclopride confirms that the NET has a large capacity for catecholamines, which is in contrast to the contention that competition between dopamine and noradrenaline for the same transporter may limit dopamine clearance from the extracellular space (Carboni et al., 1990; Gresch et al., 1995; Yamamoto and Novotney, 1998; Morón et al., 2002). On the other hand, DAT seems to play a minor role, with respect to NET, in the clearance of extracellular dopamine in mPFC. Accordingly, sparse DAT immunoreactivity has been shown in terminals and axon varicosities of dopamine neurons in mPFC (Sesack et al., 1998).

Indeed, the rise in extracellular dopamine after NET blockade should represent the amount of dopamine taken up by noradrenergic terminals from the extracellular space and, indirectly, of the amount of dopamine released from dopaminergic terminals and co-released with noradrenaline from noradrenergic terminals.

The elevation of the dopamine level demonstrates that the uptake into noradrenergic terminals plays a major role in terminating dopamine action, limiting excessive dopamine concentrations in the extracellular fluid and preventing the negative consequences of D_1_-receptor overstimulation (Zahrt et al., 1997). Mostly, the rise in dopamine after NET inhibition reconciles the apparent dissociation between the activation of dopaminergic cell firing and the lack of dopamine increase in mPFC, following the administration of typical antipsychotics, including raclopride (Gessa et al., 2000).

Due to the systemic administration of the drugs, an action at the nerve terminal level by atipamezole and raclopride is not excluded; this possibility is not alternative but complementary to an action at the cell level (Pozzi et al., 1994; Gresch et al., 1995). A limitation to this study is that microdialysis and electrophysiology were conducted under different conditions, which might have influenced the results. However, a previous study in which microdialysis and electrophysiology were performed under anesthesia indicated that haloperidol, which blocks D_2_ but not α_2_ receptors, similar to raclopride, stimulated dopamine cell firing but did not increase the extracellular dopamine level (Gessa et al., 2000). Conversely, clozapine, which blocks both D_2_ and α_2_ receptors, increased both dopamine cell firing and extracellular dopamine, similar to raclopride/atipamezole in this study. Anesthetics can affect the firing properties of dopamine neurons, meant as quantitative rather than qualitative changes. Yet, dosage adjustments for pharmacological studies are generally producing comparable effects under different anesthesia conditions (Kelland et al., 1989).

In conclusion, our results demonstrate that the combined blockade α_2_- and D_2_-receptor activates VTA cell firing and increases dopamine release in mPFC. However, dopamine release from dopamine terminals cannot be monitored by microdialysis because dopamine that escapes the synapse is taken up from extracellular fluid into noradrenergic nerve terminals. By contrast, the elevation of extracellular dopamine produced by atipamezole alone or in combination with raclopride, originates from noradrenergic terminals, independent of the firing of dopamine neurons and the amount of dopamine taken up by noradrenergic terminals.

Mounting evidence indicates that mesolimbic dopamine neurons release dopamine in the nucleus accumbens and other striatal regions in a phasic and tonic mode to control cognitive and motivational functions, respectively (Mohebi et al., 2019). Accordingly, while a fast phasic dopamine release would depend on the electrical activity of dopamine neurons, a tonic slow dopamine release has been found to be independent of the dopamine cell activity, being controlled by the cholinergic–nicotinic mechanism at the terminal level. It is appealing to speculate whether the amount of dopamine released from noradrenergic terminals, independent of the activity of dopamine neurons, might regulate motivational functions in mPFC. A better understanding of the crosstalk between dopaminergic and noradrenergic transmissions in PFC would indicate useful strategies for treating psychiatric and neurological conditions involving dopamine deficits.

## Data Availability

The original contributions presented in the study are included in the article; further inquiries can be directed to the corresponding authors.

## References

[B1] Au-Young S. M. Shen H. Yang C. R. (1999). Medial prefrontal cortical output neurons to the ventral tegmental area (VTA) and their responses to burst-patterned stimulation of the VTA: neuroanatomical and *in vivo* electrophysiological analyses. Synapse 34 (4), 245–255. 10.1002/(SICI)1098-2396(19991215)34:4<245:AID-SYN1>3.0.CO;2-D 10529719

[B2] Breier A. (1994). Clozapine and noradrenergic function: support for a novel hypothesis for superior efficacy. J. Clin. Psychiatry 55 (Suppl. B), 122–125. 7961555

[B3] Brosda J. Jantschak F. Pertz H. H. (2014). α2-Adrenoceptors are targets for antipsychotic drugs. Psychopharmacol. Berl. 231, 801–812. 10.1007/s00213-014-3459-8 24488407

[B4] Carboni E. Tanda G. L. Frau R. Di Chiara G. (1990). Blockade of the noradrenaline carrier increases extracellular dopamine concentrations in the prefrontal cortex: evidence that dopamine is taken up *in vivo* by noradrenergic terminals. J. Neurochem. 55, 1067–1070. 10.1111/j.1471-4159.1990.tb04599.x 2117046

[B5] Cedarbaum J. M. Aghajanian G. K. (1977). Catecholamine receptors on locus coeruleus neurons: pharmacological characterization. Eur. J. Pharmacol. 44, 375–385. 10.1016/0014-2999(77)90312-0 330174

[B6] Devoto P. Flore G. Longu G. Pira L. Gessa G. L. (2003). Origin of extracellular dopamine from dopamine and noradrenaline neurons in the medial prefrontal and occipital cortex. Synap. N. Y. N. 50, 200–205. 10.1002/syn.10264 14515337

[B7] Devoto P. Flore G. (2006). On the origin of cortical dopamine: is it a co-transmitter in noradrenergic neurons? Curr. Neuropharmacol. 4, 115–125. 10.2174/157015906776359559 18615131 PMC2430672

[B8] Devoto P. Flore G. Pani L. Gessa G. L. (2001). Evidence for co-release of noradrenaline and dopamine from noradrenergic neurons in the cerebral cortex. Mol. Psychiatry 6, 657–664. 10.1038/sj.mp.4000904 11673793

[B9] Devoto P. Flore G. Saba P. Scheggi S. Mulas G. Gambarana C. (2019). Noradrenergic terminals are the primary source of α2-adrenoceptor mediated dopamine release in the medial prefrontal cortex. Prog. Neuropsychopharmacol. Biol. Psychiatry 90, 97–103. 10.1016/j.pnpbp.2018.11.015 30472147

[B10] Devoto P. Sagheddu C. Santoni M. Flore G. Saba P. Pistis M. (2020). Noradrenergic source of dopamine assessed by microdialysis in the medial prefrontal cortex. Front. Pharmacol. 11, 588160. 10.3389/fphar.2020.588160 33071798 PMC7538903

[B11] Elsworth J. D. Jentsch J. D. Morrow B. A. Redmond D. E. Roth R. H. (2008). Clozapine normalizes prefrontal cortex dopamine transmission in monkeys subchronically exposed to phencyclidine. Neuropsychopharmacol. Off. Publ. Am. Coll. Neuropsychopharmacol. 33, 491–496. 10.1038/sj.npp.1301448 17507917

[B12] Frau R. Devoto P. Aroni S. Saba P. Sagheddu C. Siddi C. (2022). The potent α2-adrenoceptor antagonist RS 79948 also inhibits dopamine D2 -receptors: comparison with atipamezole and raclopride. Neuropharmacology 217, 109192. 10.1016/j.neuropharm.2022.109192 35850212

[B13] Gammon D. Cheng C. Volkovinskaia A. Baker G. B. Dursun S. M. (2021). Clozapine: why is it so uniquely effective in the treatment of a range of neuropsychiatric disorders? Biomolecules 11, 1030. 10.3390/biom11071030 34356654 PMC8301879

[B14] Gerlach J. (1991). New antipsychotics: classification, efficacy, and adverse effects. Schizophr. Bull. 17, 289–309. 10.1093/schbul/17.2.289 1715608

[B16] Gessa G. L. Devoto P. Diana M. Flore G. Melis M. Pistis M. (2000). Dissociation of haloperidol, clozapine, and olanzapine effects on electrical activity of mesocortical dopamine neurons and dopamine release in the prefrontal cortex. Neuropsychopharmacology 22 (6), 642–649. 10.1016/S0893-133X(00)00087-7 10788763

[B17] Gobert A. Rivet J. M. Cistarelli J. M. Millan M. J. (1997). Buspirone enhances duloxetine- and fluoxetine-induced increases in dialysate levels of dopamine and noradrenaline, but not serotonin, in the frontal cortex of freely moving rats. J. Neurochem. 68, 1326–1329. 10.1046/j.1471-4159.1997.68031326.x 9048781

[B18] Grace A. A. Bunney B. S. (1984). The control of firing pattern in nigral dopamine neurons: burst firing. J. Neurosci. Off. J. Soc. Neurosci. 4, 2877–2890. 10.1523/JNEUROSCI.04-11-02877.1984 PMC6564720 6150071

[B19] Gresch P. J. Sved A. F. Zigmond M. J. Finlay J. M. (1995). Local influence of endogenous norepinephrine on extracellular dopamine in rat medial prefrontal cortex. J. Neurochem. 65, 111–116. 10.1046/j.1471-4159.1995.65010111.x 7790854

[B20] Guiard B. P. El Mansari M. Merali Z. Blier P. (2008). Functional interactions between dopamine, serotonin and norepinephrine neurons: an *in-vivo* electrophysiological study in rats with monoaminergic lesions. Int. J. Neuropsychopharmacol. 11, 625–639. 10.1017/S1461145707008383 18205979

[B21] Haapalinna A. Leino T. Heinonen E. (2003). The alpha 2-adrenoceptor antagonist atipamezole potentiates anti-Parkinsonian effects and can reduce the adverse cardiovascular effects of dopaminergic drugs in rats. Naunyn Schmiedeb. Arch. Pharmacol. 368, 342–351. 10.1007/s00210-003-0827-z 14566451

[B22] Henry B. Fox S. H. Peggs D. Crossman A. R. Brotchie J. M. (1999). The alpha2-adrenergic receptor antagonist idazoxan reduces dyskinesia and enhances anti-parkinsonian actions of L-dopa in the MPTP-lesioned primate model of Parkinson’s disease. Mov. Disord. Off. J. Mov. Disord. Soc. 14, 744–753. 10.1002/1531-8257(199909)14:5<744:aid-mds1006>3.0.co;2-7 10495035

[B23] Hertel P. Fagerquist M. V. Svensson T. H. (1999a). Enhanced cortical dopamine output and antipsychotic-like effects of raclopride by alpha2 adrenoceptor blockade. Science 286, 105–107. 10.1126/science.286.5437.105 10506554

[B24] Hertel P. Nomikos G. G. Svensson T. H. (1999b). Idazoxan preferentially increases dopamine output in the rat medial prefrontal cortex at the nerve terminal level. Eur. J. Pharmacol. 371, 153–158. 10.1016/s0014-2999(99)00175-2 10357252

[B25] Ihalainen J. A. Tanila H. (2002). *In vivo* regulation of dopamine and noradrenaline release by alpha2A-adrenoceptors in the mouse prefrontal cortex. Eur. J. Neurosci. 15, 1789–1794. 10.1046/j.1460-9568.2002.02014.x 12081658

[B26] Imaki J. Mae Y. Shimizu S. Ohno Y. (2009). Therapeutic potential of alpha2 adrenoceptor antagonism for antipsychotic-induced extrapyramidal motor disorders. Neurosci. Lett. 454, 143–147. 10.1016/j.neulet.2009.03.001 19429072

[B27] Invernizzi R. W. Garattini S. (2004). Role of presynaptic alpha2-adrenoceptors in antidepressant action: recent findings from microdialysis studies. Prog. Neuropsychopharmacol. Biol. Psychiatry 28, 819–827. 10.1016/j.pnpbp.2004.05.026 15363606

[B28] Invernizzi R. W. Garavaglia C. Samanin R. (2003). The alpha 2-adrenoceptor antagonist idazoxan reverses catalepsy induced by haloperidol in rats independent of striatal dopamine release: role of serotonergic mechanisms. Neuropsychopharmacol. Off. Publ. Am. Coll. Neuropsychopharmacol. 28, 872–879. 10.1038/sj.npp.1300119 12644843

[B29] Kalkman H. O. Loetscher E. (2003). Alpha2C-Adrenoceptor blockade by clozapine and other antipsychotic drugs. Eur. J. Pharmacol. 462, 33–40. 10.1016/s0014-2999(03)01308-6 12591093

[B30] Kapur S. Remington G. (2001). Atypical antipsychotics: new directions and new challenges in the treatment of schizophrenia. Annu. Rev. Med. 52, 503–517. 10.1146/annurev.med.52.1.503 11160792

[B31] Kelland M. D. Freeman A. S. Chiodo L. A. (1989). Chloral hydrate anesthesia alters the responsiveness of identified midbrain dopamine neurons to dopamine agonist administration. Synapse 3 (1), 30–37. 10.1002/syn.890030105 2919369

[B32] Khokhar J. Y. Henricks A. M. Sullivan E. D. K. Green A. I. (2018). Unique effects of clozapine: a pharmacological perspective. Adv. Pharmacol. San. Diego Calif. 82, 137–162. 10.1016/bs.apha.2017.09.009 PMC7197512 29413518

[B33] Langer S. Z. (2015). α2-Adrenoceptors in the treatment of major neuropsychiatric disorders. Trends Pharmacol. Sci. 36, 196–202. 10.1016/j.tips.2015.02.006 25771972

[B34] Litman R. E. Su T. P. Potter W. Z. Hong W. W. Pickar D. (1996). Idazoxan and response to typical neuroleptics in treatment-resistant schizophrenia. Comparison with the atypical neuroleptic, clozapine. Br. J. Psychiatry J. Ment. Sci. 168, 571–579. 10.1192/bjp.168.5.571 8733795

[B35] Lohani S. Martig A. K. Underhill S. M. DeFrancesco A. Roberts M. J. Rinaman L. (2018). Burst activation of dopamine neurons produces prolonged post-burst availability of actively released dopamine. Neuropsychopharmacology 43 (10), 2083–2092. 10.1038/s41386-018-0088-7 29795245 PMC6098082

[B36] MacKinnon A. C. Brown C. M. Spedding M. Kilpatrick A. T. (1989). [3H]-idazoxan binds with high affinity to two sites on hamster adipocytes: an alpha 2-adrenoceptor and a non-adrenoceptor site. Br. J. Pharmacol. 98, 1143–1150. 10.1111/j.1476-5381.1989.tb12658.x 2558757 PMC1854795

[B37] Marcus M. M. Wiker C. Frånberg O. Konradsson-Geuken A. Langlois X. Jardemark K. (2010). Adjunctive alpha2-adrenoceptor blockade enhances the antipsychotic-like effect of risperidone and facilitates cortical dopaminergic and glutamatergic, NMDA receptor-mediated transmission. Int. J. Neuropsychopharmacol. 13, 891–903. 10.1017/S1461145709990794 19835668

[B38] Masana M. Bortolozzi A. Artigas F. (2011). Selective enhancement of mesocortical dopaminergic transmission by noradrenergic drugs: therapeutic opportunities in schizophrenia. Int. J. Neuropsychopharmacol. 14, 53–68. 10.1017/S1461145710000908 20701825

[B39] Mejias-Aponte C. A. (2016). Specificity and impact of adrenergic projections to the midbrain dopamine system. Brain Res. 1641, 258–273. 10.1016/j.brainres.2016.01.036 26820641 PMC4879068

[B40] Millan M. J. Newman-Tancredi A. Audinot V. Cussac D. Lejeune F. Nicolas J. P. (2000). Agonist and antagonist actions of yohimbine as compared to fluparoxan at alpha(2)-adrenergic receptors (AR)s, serotonin (5-HT)(1A), 5-HT(1B), 5-HT(1D) and dopamine D(2) and D(3) receptors. Significance for the modulation of frontocortical monoaminergic transmission and depressive states. Synap. N. Y. N. 35, 79–95. 10.1002/(SICI)1098-2396(200002)35:2<79:AID-SYN1>3.0.CO;2-X 10611634

[B41] Mohebi A. Pettibone J. R. Hamid A. A. Wong J-M. T. Vinson L. T. Patriarchi T. (2019). Dissociable dopamine dynamics for learning and motivation. Nature 570, 65–70. 10.1038/s41586-019-1235-y 31118513 PMC6555489

[B42] Morón J. A. Brockington A. Wise R. A. Rocha B. A. Hope B. T. (2002). Dopamine uptake through the norepinephrine transporter in brain regions with low levels of the dopamine transporter: evidence from knock-out mouse lines. J. Neurosci. Off. J. Soc. Neurosci. 22, 389–395. 10.1523/JNEUROSCI.22-02-00389.2002 PMC6758674 11784783

[B43] Muntoni A. L. Pillolla G. Melis M. Perra S. Gessa G. L. Pistis M. (2006). Cannabinoids modulate spontaneous neuronal activity and evoked inhibition of locus coeruleus noradrenergic neurons. Eur. J. Neurosci. 23, 2385–2394. 10.1111/j.1460-9568.2006.04759.x 16706846

[B44] Newman-Tancredi A. Chaput C. Gavaudan S. Verrièle L. Millan M. J. (1998). Agonist and antagonist actions of (-)pindolol at recombinant, human serotonin1A (5-HT1A) receptors. Neuropsychopharmacol. Off. Publ. Am. Coll. Neuropsychopharmacol. 18, 395–398. 10.1016/S0893-133X(97)00169-3 9536453

[B45] Ortega J. E. Fernández-Pastor B. Callado L. F. Meana J. J. (2010). *In vivo* potentiation of reboxetine and citalopram effect on extracellular noradrenaline in rat brain by α2-adrenoceptor antagonism. Eur. Neuropsychopharmacol. J. Eur. Coll. Neuropsychopharmacol. 20, 813–822. 10.1016/j.euroneuro.2010.07.008 20813509

[B46] Ostock C. Y. Hallmark J. Palumbo N. Bhide N. Conti M. George J. A. (2015). Modulation of L-DOPA's antiparkinsonian and dyskinetic effects by α2-noradrenergic receptors within the locus coeruleus. Neuropharmacology 95, 215–225. 10.1016/j.neuropharm.2015.03.008 25817388 PMC4466080

[B47] Park J. W. Bhimani R. V. Park J. (2017). Noradrenergic modulation of dopamine transmission evoked by electrical stimulation of the locus coeruleus in the rat brain. ACS Chem. Neurosci. 8, 1913–1924. 10.1021/acschemneuro.7b00078 28594540

[B48] Pozzi L. Invernizzi R. Cervo L. Vallebuona F. Samanin R. (1994). Evidence that extracellular concentrations of dopamine are regulated by noradrenergic neurons in the frontal cortex of rats. J. Neurochem. 63, 195–200. 10.1046/j.1471-4159.1994.63010195.x 8207428

[B49] Rascol O. Arnulf I. Peyro-Saint Paul H. Brefel-Courbon C. Vidailhet M. Thalamas C. (2001). Idazoxan, an alpha-2 antagonist, and L-DOPA-induced dyskinesias in patients with Parkinson’s disease. Mov. Disord. Off. J. Mov. Disord. Soc. 16, 708–713. 10.1002/mds.1143 11481696

[B50] Savola J-M. Hill M. Engstrom M. Merivuori H. Wurster S. McGuire S. G. (2003). Fipamezole (JP-1730) is a potent alpha2 adrenergic receptor antagonist that reduces levodopa-induced dyskinesia in the MPTP-lesioned primate model of Parkinson’s disease. Mov. Disord. Off. J. Mov. Disord. Soc. 18, 872–883. 10.1002/mds.10464 12889076

[B51] Scatton B. Zivkovic B. Dedek J. (1980). Antidopaminergic properties of yohimbine. J. Pharmacol. Exp. Ther. 215, 494–499. 7192314

[B52] Sesack S. R. Hawrylak V. A. Matus C. Guido M. A. Levey A. I. (1998). Dopamine axon varicosities in the prelimbic division of the rat prefrontal cortex exhibit sparse immunoreactivity for the dopamine transporter. J. Neurosci. 18 (7), 2697–2708. 10.1523/JNEUROSCI.18-07-02697.1998 9502827 PMC6793120

[B53] Svensson T. H. (2003). Alpha-adrenoceptor modulation hypothesis of antipsychotic atypicality. Prog. Neuropsychopharmacol. Biol. Psychiatry 27, 1145–1158. 10.1016/j.pnpbp.2003.09.009 14642973

[B54] The Rat Brain (2022). The rat brain in stereotaxic coordinates - 6th edition. Available at: https://www.elsevier.com/books/the-rat-brain-in-stereotaxic-coordinates/paxinos/978-0-12-374121-9 (Accessed December 7, 2022).

[B56] Wadenberg M-L. Wiker C. Svensson T. H. (2007). Enhanced efficacy of both typical and atypical antipsychotic drugs by adjunctive alpha2 adrenoceptor blockade: experimental evidence. Int. J. Neuropsychopharmacol. 10, 191–202. 10.1017/S1461145706006638 16707032

[B57] Wallace L. J. Traeger J. S. (2012). Dopac distribution and regulation in striatal dopaminergic varicosities and extracellular space. Synap. N. Y. N. 66, 160–173. 10.1002/syn.20996 21987292

[B58] Yamamoto B. K. Novotney S. (1998). Regulation of extracellular dopamine by the norepinephrine transporter. J. Neurochem. 71, 274–280. 10.1046/j.1471-4159.1998.71010274.x 9648875

[B59] Zahrt J. Taylor J. R. Mathew R. G. Arnsten A. F. (1997). Supranormal stimulation of D1 dopamine receptors in the rodent prefrontal cortex impairs spatial working memory performance. J. Neurosci. Off. J. Soc. Neurosci. 17, 8528–8535. 10.1523/JNEUROSCI.17-21-08528.1997 PMC6573725 9334425

